# Biliary Calculi Imitating Organic Affection of the Stomach

**Published:** 1831

**Authors:** 


### XLVIII.

### BILIARY CALCULI IMITATING ORGANIC AFFECTION OF THE STOMACH. By M. BRICHETEAU.

*Case.* A BOOKSELLER, aged 67 years, of good constitution, but of bilious temperament, had led an active life, chequered, of late, with many reverses of fortune, and those of the sombre cast. In the month of April, 1828, he experienced, for the first time, acute pains in the umbilical region, for which fomentations and leeches were employed, and with temporary relief. In the night of the 19th, 20th, he was in great sufferings for five or six hours; but they were relieved by liniments containing a considerable proportion of laudanum. A day or two afterwards M. Bricheteau carefully examined the abdomen, and found that the epigastrium was very tender on pressure, and offered unnatural resistance in the region of the pylorus. This exploration induced the author to fear that there was some organic affection of that part. These fears were augmented when the patient informed the Doctor, that he experienced a sense of weight in his stomach after food, succeeded by nausea, and even vomiting. These vomitings occurred at various intervals after eating, and often consisted of dark and viscid matter. Various means were tried, with little effect; and the patient continued to suffer till the month of July, 1829, when an unusually violent attack caused M. Bricheteau to be again summoned. The tenderness on pressure was now more in the right hypochondre, and the pain seemed to radiate thence to different parts of the abdomen. M. B. now began to suspect that the cause of the sufferings might probably be biliary calculi. His diagnosis this time was confirmed by the discharge of a great number of biliary concretions by stool, a few days afterwards. They were analyzed by M. Chevallier. The accessions, however, returned once in the week for eight or nine weeks in succession; and each time there was a discharge of biliary calculi. The attacks then ceased entirely till the month of June, 1830, when he suffered very severely in the usual way, and a considerable number of calculi were discharged by stool. After this his health was re-established completely.—JOURN. COMPLEMENT.

It is not a little remarkable that no yellowness of the skin is mentioned by the author in any of these numerous attacks of biliary calculi. We have very little doubt that this phenomena presented itself, though unnoticed by the French narrator.

